# A mixed-methods analysis of personal protective equipment used in Lassa fever treatment centres in Nigeria

**DOI:** 10.1016/j.infpip.2021.100168

**Published:** 2021-08-03

**Authors:** Andrew Holt, Emilio Hornsey, Anna C. Seale, Hana Rohan, Daniel G. Bausch, Chikwe Ihekweazu, Tochi Okwor

**Affiliations:** a London School of Hygiene & Tropical Medicine, London, UK; b UK Public Health Rapid Support Team, London, UK; c Public Health England, UK; d Nigeria Center for Disease Control, Abuja, Nigeria

**Keywords:** Lassa fever, Personal protective equipment, Infection prevention and control, Risk assessment

## Abstract

**Background:**

Lassa fever (LF) is a viral haemorrhagic fever endemic in West Africa. Lassa virus is maintained in and spread to humans from rodents, with occasional secondary human-to-human transmission. Present recommendations for personal protective equipment (PPE) for care of patients with LF generally follow those for filovirus diseases. However, the need for such high-level PPE for LF, which is thought to be considerably less transmissible between humans than filoviruses, is unclear.

**Aim:**

In Nigerian Lassa Treatment Centres (LTCs) we aimed to describe current PPE practices, identify barriers and facilitators to implementation of existing guidance, and assess healthcare workers' understanding. This would inform the development of future PPE guidelines for LF.

**Methods:**

We performed a mixed-methods study, including short cross-sectional surveys of PPE used in LTCs, observations of practice, and in-depth interviews with key informants. We described the quantitative data and we conducted a thematic analysis of qualitative data.

**Findings:**

Our survey of 74 HCWs found that approximately half reported problems with recommended PPE. In three LTCs PPE was used highly variably. Full PPE, as recommended in Nigeria CDC guidelines, was used in less than a quarter (21%) of interactions. In-depth interviews suggested this was based on availability and HCWs' own risk assessments.

**Conclusion:**

Without specific guidance on Lassa, the current approach is both resource and labour-intensive, where these are both limited. This has led to low adherence by health care workers, whose own experience indicates lower risk. The evidence-base to inform PPE required for LF must be improved to inform a more tailored approach.

## Introduction

Lassa fever (LF) is an acute febrile illness whose effects range from asymptomatic infection to serious multiorgan dysfunction, haemorrhage, neurological manifestations and death [1,2]. The disease, caused by Lassa virus (family *Arenaviridae*), is endemic across large areas of West Africa, where it is maintained in and spread to humans primarily in the “multimammate rat” *Mastomys natalensis*) [3], with occasional secondary person-to-person transmission, particularly in hospitals with inadequate infection prevention and control (IPC) [4,5].

Current recommendations for personal protective equipment (PPE) for care of persons with LF generally follow those for viral haemorrhagic fever (VHF) caused by Ebola and Marburg viruses (family *Filoviridae*) [6]. However, the infectivity of Lassa virus is thought to be considerably less than the filoviruses, [3,[7], [8], [9]], calling into question whether such high-level PPE, which poses considerable logistic and financial challenges, is truly indicated. Furthermore, currently recommended PPE for LF allows for little evaporative cooling, severely limiting HCWs' ability to provide care, especially in the generally hot climates where LF is endemic.

## Background

Much of the published literature on PPE for LF consists of case reports from imported cases to high-income settings (To see results of a systematic literature review conducted in advance of this study please refer to Appendix 1). These cases have resulted in no secondary clinical infections thus far, and only two reported seroconversions [10]^,^ even when LF was not suspected and standard precautions were employed [11]. A recent report on an imported case in Germany concluded: “Use of PPE including gloves, gowns, masks and goggles is effective in prevention of transmission.” [12]. Outbreak reports in health care settings from low- and middle-income settings have all occurred in non-specialist clinical settings [13,14] and have often related to invasive procedures such as surgery [15]. No investigations of PPE requirements for LF in endemic areas for the disease have been reported.

Recognising these challenges, the World Health Organization recently set out preferred product characteristics for PPE for LF, emphasising the need to improve current ensembles [16]. The World Health Organization and the Cochrane review [17] both highlighted significant gaps in knowledge around PPE design, preferred types of PPE and best practice for donning and doffing.

To explore barriers and facilitators for implementation of PPE for LF, and to inform future guidelines, we explored current practices in three Lassa Treatment Centres (LTCs) in Nigeria.

## Methods

### Research design

We used a mixed-methods approach, utilising a triangulated design [18]: 1) a short cross-sectional survey for HCWs, 2) observations of IPC practices in LTCs, and 3) in-depth interviews with key informants. The cross-sectional survey of HCWs' perceptions of risk and protective measures for LF was a structured, interviewer-administered questionnaire. We used a broad convenience sampling technique to recruit all HCWs who were working during each visit to three LTCs. We defined HCWs as doctors, nurses, and others including laboratory technicians, social workers, hygienists, and care assistants in accordance with standard classification [19,20]. Observations of IPC practices in the LTCs comprised a direct, overt visual audit of practice, with a checklist based on Nigeria Center for Disease Control guidelines [21].

We collected data using Open Data Kit forms designed by the team and piloted at Federal Teaching Hospital Abakaliki (FETHA). We performed observations and administered surveys at three LTCs; Irrua Specialist Teaching Hospital (ISTH) in Edo state, FETHA in Ebonyi state, and Federal Medical Centre Owo (FMC Owo) in Ondo state. These sites are in endemic areas and care for over 80% of all confirmed LF cases in Nigeria [14]. We spent five days at ISTH, three at FETHA, and four at FMC Owo. All elements of the data collection were completed concurrently while we were at each site. We conducted 19 in-depth interviews, including nine at ISTH and five each at FETHA and at Owo. Some interviewees worked across sites or at state or national levels. Interviewees were senior staff who had experience with LF across a range of domains, including clinical care, policy development, and public health. We used a topic guide to direct the conversation to preidentified themes (see Appendix 4 for the Topic Guide), with free discussion. Informants consisted of policy makers and clinicians identified through purposive sampling and progressive snowball sampling. Interviews were recorded and conducted in English. We transcribed and anonymised the interviews into Nvivo (v12). A systematic form of thematic analysis was conducted [18,22], data were coded into the preidentified themes and subthemes, as well as novel codes that arose from the data. Transcripts and coding were independently reviewed by another team member. Transcripts were then revisited to ensure coding consistency, and differences resolved through team discussion and developing consensus. Due to time restrictions at the sites, member checking of transcripts was not a feasible process.

We analysed data using Stata SE14® (descriptive and comparative analyses). We coded survey data (free text data) and in-depth interviews using Nvivo 12, taking a thematic approach that incorporated both inductive and deductive components [18,22]. We disaggregated quantitative data by location, demographic variables, training and experience.

## Findings

### Cross-sectional survey

**Figure 1:**
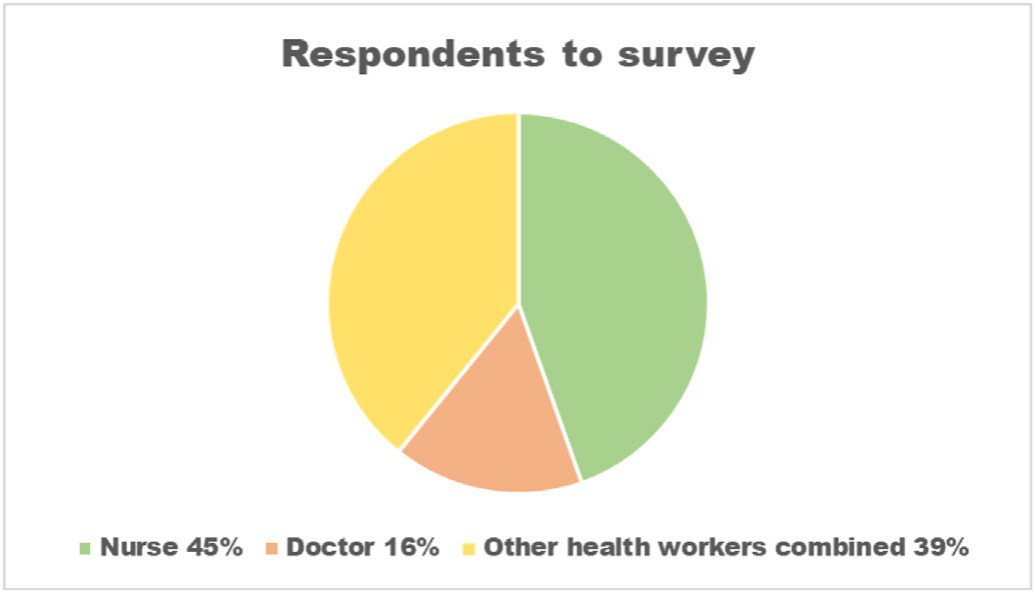
Respondents to survey.

**Figure 2:**
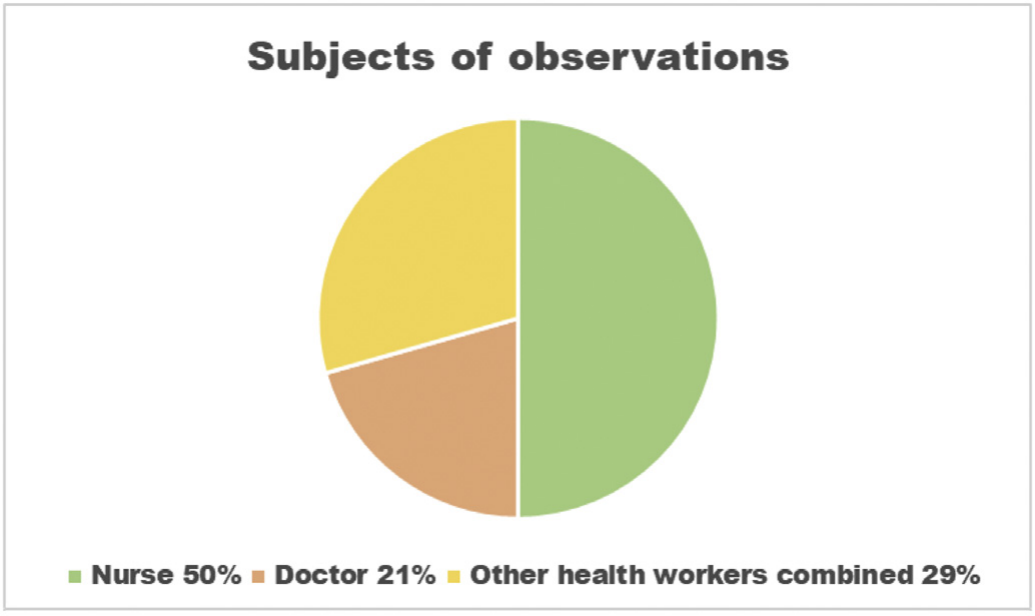
Subjects of observations.

We surveyed 74/106 (70%) of all HCWs, 31 at ISTH, 15 at FETHA and 28 at Owo, of whom 47 (64%) were female and the median age was 36 years (range 22–58 years) (Figure 2 in Appendix 2). HCWs were comprised of 33 (45%) nurses, 12 (16%) doctors, and 29 (39%) consisted of a range of other health workers (Figure 1).

Other health workers a range of non-professional and professional staff such as hygienists (who are responsible for tasks such as waste management and disinfection of reusable PPE), Cleaners, Porters, Social Workers and medical specialists.

While 62 (84%) HCWs replied that PPE was always available at their LTC, they noted frequent stock outs for individual items, such as gloves (57, 77%) and cover-all suits (51, 69%).

HCWs reported subtle variations of PPE use between sites, for instance, at both FETHA and Owo, there was higher reported use of N95 masks, hoods, and boots while at ISTH there was higher reported use of surgical as opposed to N95 masks, a preference of surgical caps over hoods, and slip-on shoes rather than boots (See Figure 1 Appendix 2).

Half of all HCWs reported problems wearing the recommended PPE ensemble, especially cover-all suit (45% reported difficulties); 13 (87%) HCWs reported issues at FETHA, where a thicker yellow suit is provided, versus only 11 (36%) at ISTH, where a lighter version and locally made alternatives are available. Problems reported included overheating, dehydration and restriction of movement. Gloves were the next most problematic, with 18 (24%) HCWs across sites reporting ripping or perforating, and perceived variable quality. Next was goggles for which 11 (15%) respondents reported fogging and reduced visibility. Although FETHA had been supplied with anti-fog spray by their NGO partner, HCWs still reported problems. HCWs at Owo preferred visors or abandoning goggles altogether and risk working with no eye protection to avoid this issue. However, when worn, HCW confidence in the protection provided by the current PPE ensemble was high, with 32 (43%) reporting that they felt “Always protected – 100% of the time” and 42% reporting “Effective protection – between 75–100% of the time”; the remaining 15% thought PPE was less than 75% effective, with two (3%) HCWs saying protection was less than 50%.

Respondents reported various factors affecting choice of PPE, with the main drivers being risk of exposure to bodily fluids (34, 46%), type of procedure carried out (36, 49%), severity of patient illness (22, 30%) and availability of PPE (17, 23%). (Full results of the survey are available at Appendix 2)

### Observations on PPE in practice

We completed 34 observations, 26 (76%) at ISTH and 8 (24%) at Owo. No observations were possible at FETHA since there were no cases there during the period of study. Observations were with nurses 17 (50%), doctors 7 (21%) and a range of other health workers 10 (29%) and were classified by the rationale for entering the high-risk zone (e.g. giving medications, taking blood samples or decontamination and cleaning) (Figure 2).

We observed the process of PPE donning by HCWs entering the high-risk zone. While most (91%) donning occurred within the designated area, that figure fell to 73% for doffing. PPE was disposed of in the appropriate waste stream 76% of the time. The full ensemble as prescribed by Nigeria Centre for Disease Control guidelines [21] was worn in only 20% of interactions. Some components of the PPE were used almost universally while use of others was substantially varied between sites and HCWs. For example, scrubs were worn under PPE during 91% of observations, with only 3 HCWs wearing their own clothes underneath PPE (all doctors). Gloves were worn in 97% of interactions, but double gloves only in 62%. At Owo, all HCWs wore almost identical ensembles – scrubs, cover-all suits, boots, N95 masks, aprons and double gloves. The only variation observed was whether HCWs wore goggles or a visor. By contrast, at ISTH, use of surgical masks and surgical caps was high but use of double gloves was low (observed in only 54% of interactions at that site). At ISTH there was mixed use of cover-all suits and gowns, and slip-on shoes were predominantly used, instead of boots.

Comparing cross-sectional survey data and observations, there were seven items of PPE for which we observed a difference between reported and observed use. Of these only scrubs had higher observed-to-reported use. Items reported to have much higher usage than observed were double gloves, aprons, goggles, N95 masks, hoods and boots. All 21% of HCWs who wore goggles, N95 mask and boots were based at one site (to see full data from the observations please refer to Appendix 3).

### Interviews with key informants

The analysis revealed consistency across sites relating to how availability of PPE, risk assessment, length of admission, patient symptoms and medical procedures affected protective measures taken. A range of themes were identified and a number of these are explored and interpreted below, along with illustrative quotes from LTC clinicians. An overview of all the major themes identified is in Appendix 4.

#### Theme: availability and quality of PPE

Many respondents discussed a general lack of availability of PPE in LTCs and cited a variety of reasons. Unpredictable patient loads were perceived to threaten the supply chain and add to difficulties planning for supplies. At some sites an NGO had helped with availability issues. There was also discussion of how people respond to and resolve these issues.

‘The supply is not constant, so that is why we are not supplying [wearing] it. We know that it is in the guidelines, but we don't have it.’

‘Sometimes we don't have PPEs and, whether we are here, if we don't have PPE to use we can't say we are not going to attend to the patient.’

Many respondents talked about availability issues that arose not from total absence of supply, but because of incorrect products being provided, with poor-quality PPE or wrong sizes sometimes being supplied. In particular, there were quality concerns about risk of breach, inappropriate materials and poor fitting.

‘Some of the PPE [cover-alls] are not appropriate. Some are too small, like I remember some of the ones we wore in the past and part of your feet is sticking out or some that the gloves are too small or incomplete and then some that the gloves are poor quality, or the PPEs are poor quality.’

Many respondents remarked that PPE guidance and practices had changed over time, especially in regard to the 2013–16 West Africa Ebola virus disease epidemic, with the perception that PPE guidelines had become stricter and more extensive as a result of that outbreak. Respondents questioned whether this was appropriate.

‘We didn't know what was called PPE, I was using my ward coat and did not use all those things and people were surprised that I didn't come down with it.’

‘During the Ebola outbreak, because you don't know which is Lassa and which is Ebola, because somebody's come in with fever and you think it might be other forms of viral haemorrhagic fever, so you want to put on the full PPE.’

‘But after 2014, that's when I started seeing more generous PPE, which actually came from Ebola scare, and the first impression that Lassa was like Ebola. The dividing line is wide. Lassa is not Ebola.’

Some respondents noted that supply of PPE is also an issue outside of LTCs because LF patients are sometimes cared for in non-specialist clinical areas due to delayed diagnosis or atypical presentation. PPE was used inconsistently in these areas.

‘In accident and emergency the only difference is that we don't wear gowns, there is no gown.’

‘In the ward then we are not actually having PPE. It was more of using your hand gloves and your face masks.’

#### Personal risk assessment

Many respondents spontaneously discussed their approaches to HCW personal risk assessment, with *procedures*, *patient clinical presentation,* and *clinical area* being the main factors that respondents used to assess their own risk. Different tasks or procedures were perceived by respondents as having higher or lower risk. Surgical interventions were generally seen to carry the highest risk of infection. Participants drew largely on empirical knowledge as opposed to formal training to make their assessment.

‘This full PPE is not truly necessary for all patients for all procedures. For instance, if I am just going in to serve medication or serve food, I don't need to wear the full PPE because whether people like it or not Lassa is not as virulent as Ebola.’

‘Unfortunately, epidemics among the HCWs were coming from the surgical department who really could not detect them until they had issues controlling bleeding and such and at which time the viremia was really high and the prognosis was poor. Over time we have had doctors and nurses who have been infected. Most of them survive, but we have lost a few.’

When assessing risk, many respondents categorised patients, with frequent use of terminology of ‘wet’ or ‘dry’. Other respondents used terms such as ‘very bad’, ‘severe disease’, ‘classic’, or ‘highly toxic’. Most participants used such descriptors to explain how they assessed risk.

‘Wet symptoms were defined by diarrhoea, vomiting, bleeding, coughing. If they have any of those ones. I say give them single isolation, while those ones that have dry symptoms we could cohort them because, from our experience, we don't think the transmission is very high among those.’

‘Usually some patients are wet, and some patients are dry. When we look at the patient and we see that they are wet we have to wear the full PPE, but for patients that are not wet we just have to make sure that we are wearing the N95, that we are wearing the gloves of course, and the gown, and our scrubs before the gown, and we carefully take our samples.’

‘The full overall PPE is very important when the patients are wet, but when you've been managing patients the symptom are abated they are not wet, you use the light PPE.’

In addition to “wet” and “dry” status, respondents discussed other clinical factors that they perceived to affect potential risk of transmission. Patients with neurological complications were perceived to present a higher risk because their behaviour could be unpredictable.

‘When the person is also having seizure, the person will not cooperate. That is a dangerous patient, because I remember that I had something like prick (needlestick) when the patient was not cooperating.’

‘Maybe the patient is confused and irrational. You don't know what is going to happen, how they are going to behave. He might take the syringe off me and just (mimes a needle prick).’

Longer admission time, presumably as a proxy indicator of patients entering convalescent stages, was often understood to reduce risk. Accordingly, respondents reported assessing risk as lower for these patients whose transmissibility was perceived to have waned.

‘We should have different shades of PPE. The reason is that, if the patient has been on the ward for at least 5–10 days, you don't need to wear the complete PPE. For that patient you could wear a surgical apron that is properly covered, wear your double hand gloves, wear your boots to see the patients, so you can reduce the quantity of PPE you use on the patient.’

## Discussion

VHF is a syndromic diagnosis of a disease that can be caused by over 30 different viruses from four taxonomic families of virus. These families and viruses may possess drastically different biological properties. Guidelines on PPE for VHF clinical care have generally been developed with filovirus outbreaks in mind and emphasised adherence to strict PPE ensembles, including detailed procedures for donning and doffing [21,23,24]. In the setting of an endemic disease, such as LF in Nigeria and across most of West Africa, in which costs and long-term maintenance of appropriate IPC practices must be maintained indefinitely, HCW approach to the use of PPE may vary considerably.

Indeed we found HCWs in Nigeria to be taking a much more nuanced approach to PPE. In the absence of specific IPC guidelines for LF, HCWs appear to be mitigating risk based on product availability, balancing local norms and interpretation of guidance documents, and personal preference. Risk assessments by HCWs were pragmatic and often included severity of clinical symptoms and type of clinical procedure, either of which could increase exposure to blood or bodily fluids and risk of transmission. While these approaches may be pragmatic and logical, such extreme variation in interpretation and practice, with reliance on each individual's personal judgement, may entail risks for the HCW who judges incorrectly. It is also important to note the practical difficulties with availability of PPE. Were standard VHF guidelines implemented in full, the resource demands, already substantial, would likely be unsustainable.

We recognise numerous limitations to our study. The findings from three LTCs in Nigeria may not be generalisable to other hospitals or wards in Nigeria or elsewhere, although we feel that it is highly probable that HCWs across West Africa face similar challenges and limitations with regard to PPE for LF and may well adopt similar coping strategies. While there is little comparable data from LTCs specifically, other clinical settings in Nigeria (such as TB or Obstetric units) have recently reported similar PPE availability issues [25,26] along with other locally developed strategies to mitigate the implications of this, such as asking family members to procure PPE or HCWs changing their behaviour when providing patient care. This reinforces the issue of PPE insufficiency in other Nigerian health settings. Our focus was on HCWs, and did not include family care givers, who often care for patients in LTCs, supported with variable levels of PPE and training. There is presently no universal guidance for family care givers.

While we worked to reduce influence of social desirability bias and observer effects during the study, this may have occurred, leading to over-estimation of use of PPE. A clear example of participant reactivity occurred when a nurse prepared to enter the high-risk zone wearing scrubs, slip-on shoes and a mask and, upon observing a member of the study team, returned to the donning area to dress in a cover-all suit and gloves. We triangulated data wherever possible using multiple data sources to minimise these effects. The qualitative data helps provide some context for the variation we observed in practice and in response to survey questions. There may also have been misclassification of critical concepts during surveys or key informant interviews, as the work was conducted in English, in which not all HCWs were fluent.

The timing of the study may also have influenced findings; it was conducted in August, a time of year when the incidence of LF is typically lower, which limited the number of cases for which we could make observations in practice. In addition, while several new and acutely unwell patients were admitted during the study, none had haemorrhagic or ‘wet’ symptoms. It may be that, if such patients were admitted, PPE selection and HCW practices would have been different.

The present guidance for PPE use for LF in West Africa, based on experience with filoviruses (and still controversial even for these viruses), is neither evidence-based nor sustainable. First and foremost, research is required to better characterize the true nature and risk of person-to-person transmission of Lassa virus, as opposed to other causes of VHF. Secondly, policy and IPC guidance must consider the cost and logistical challenges to year-round maintenance of LTCs in endemic areas, making sure that such guidance is implementable within the confines of available resources. Safe disposal and limiting environmental contamination through waste are other important factors to consider. Innovative approaches for PPE for all VHFs are needed to accomplish these goals. Recognising this, an expert Working Group on PPE Innovations has been established by the World Health Organization, although the focus to date has been primarily on the filoviruses. We also recognise that PPE is only one element of effective IPC and that any risk assessment or improvement plan should be fully integrated with other programmatic, engineering and administrative controls [27] and not addressed as a single control measure in isolation.

The HCWs in this study typically work with severely ill patients in the context of a transmissible disease. It is imperative that efforts are made to enable them to work as comfortably and safely as possible. Some respondents in this study reported that a more nuanced and limited use of PPE has not been implicated in HCW LF transmission in LTCs, in that there had been no reported clinical infections among staff.

However, we do not yet know if HCWs are at significantly greater risk of contracting disease (or sub clinical infection identified through serological investigation) if they work in LTCs or other non-specialist health settings, in comparison to the general population. The only serological study so far conducted in an LTC in an endemic country (Sierra Leone) was limited in scope but HCWs did not show increased prevalence in relation to the general population [28]. There have however been outbreaks of nosocomial Lassa reported following surgical or obstetric procedures in general health facilities and these have claimed the lives of both HCWs and patients [4,13,15,29].

There are certainly many financial and operational considerations related to use of PPE and significant investment and product development are required to determine the appropriate standards and level of PPE [30] to protect HCWs. However, improved evidence on specific risk of transmission of LF in clinical settings could encourage less extensive use of PPE in particular contexts, facilitate HCW work, and allow resources to be directed more efficiently.

## Credit author statement

Andrew Holt: Methodology; Software; Formal analysis; Investigation; Data curation; Writing original draft.

Emilio Hornsey: Methodology; Formal analysis; Investigation; Writing original draft; Project administration.

Anna C Seale: Writing review and editing; Supervision.

Hana Rohan: Methodology; Writing review and editing.

Daniel G Bausch: Conceptualisation; Supervision; Writing review and editing.

Chikwe Ihekweazu: Conceptualisation; Supervision; Writing review and editing.

Tochi Okwor: Methodology; Formal analysis; Resources; Investigation; Writing review and editing; Supervision.
