# Supplementary material for: A mixed-methods analysis of personal protective equipment used in Lassa fever treatment centres in Nigeria
Source: Infect Prev Pract. 2021 Aug 3;3(3):100168. doi: 10.1016/j.infpip.2021.100168 (PMC8367797; doi:10.1016/j.infpip.2021.100168)
Supplement: Multimedia component 1 [file mmc1.docx]

**Appendix 1**

**Systematic review search strategy and results summarised by article type in tables below**

**Search Terms for Systematic Review**

Databases:

Global Health <1910 to 2019 Week 22>,

Embase <1974 to 2019 June 07>,

Ovid MEDLINE(R) and Epub Ahead of Print, In-Process & Other Non-Indexed Citations, Daily and Versions(R) <1946 to June 07, 2019>

Search Strategy:
--------------------------------------------------------------------------------
1     Lassa fever/ (1960)
2     Lassa virus/ (2359)
3     exp arenaviridae/ (11174)
4     1 or 2 or 3 (11983)
5     exp patient isolation/ or exp isolation hospital/ or exp isolation facility/ or exp isolation procedure/ or exp isolation/ (354396)
6     exp health care personnel/ or exp infection control/ or exp hospital infection/ (1681290)
7     (PPE or IPC).mp. (17290)
8    (protecti* adj3 (equipment or cloth* or personal or basic)).mp. (45414)
9    (infection adj3 (prevention or control)).mp. (232351)
10     (barrier adj3 (nursing or precautions or isolation or protecti*)).mp. (7945)
11     5 or 6 or 7 or 8 or 9 or 10 (2181798)
12     4 and 11 (899)

> Searches 7, 8, 9 and 10; ‘mp’ = title, abstract, heading word, drug trade name, original title, device manufacturer, drug manufacturer, device trade name, keyword, floating subheading word, candidate term word
