# Supplementary material for: A mixed-methods analysis of personal protective equipment used in Lassa fever treatment centres in Nigeria
Source: Infect Prev Pract. 2021 Aug 3;3(3):100168. doi: 10.1016/j.infpip.2021.100168 (PMC8367797; doi:10.1016/j.infpip.2021.100168)
Supplement: Multimedia component 2 [file mmc2.docx]

**Appendix 2–Results of Cross sectional survey presented by location, gender and other question parameters.**

**Figure 1:** Self reported PPE Used by HCWs - broken down by Lassa Treatment Centre

Number of responses

Number of responses

Number of responses

**Figure 2:** Self reported PPE Needed by HCWs to work safely – broken down by Lassa Treatment Centre

Number of responses

Number of responses

Number of responses

Item of PPE

**Table 1:** Reported PPE used by health workers at all sites, broken down by gender

| **Reported PPE Used** | **Male** |  | **Female** |  | **Male %** | **Female %** |
| --- | --- | --- | --- | --- | --- | --- |
|  | **Use** | **Don’t Use** | **Use** | **Don’t Use** |  |  |
| Glove | 27 | 0 | 47 | 0 | 100% | 100% |
| Double glove | 25 | 2 | 44 | 3 | 93% | 94% |
| **Heavy duty gloves** | **16** | **11** | **37** | **10** | **59%** | **78%** |
| Apron | 22 | 5 | 39 | 8 | 81% | 83% |
| Gown | 25 | 2 | 40 | 7 | 93% | 85% |
| Cover-all suit | 19 | 8 | 32 | 15 | 70% | 68% |
| Scrubs | 20 | 7 | 33 | 14 | 74% | 70% |
| Visor | 4 | 23 | 9 | 38 | 15% | 19% |
| Goggles | 23 | 4 | 39 | 8 | 85% | 83% |
| N95 Mask | 14 | 13 | 29 | 18 | 52% | 62% |
| **Surgical Mask** | **13** | **14** | **31** | **16** | **48.1%** | **66%** |
| Hood | 17 | 10 | 29 | 18 | 63% | 62% |
| **Surgical Cap** | **14** | **13** | **35** | **12** | **52%** | **74%** |
| Boots | 22 | 5 | 39 | 8 | 81% | 82% |
| Slip on shoes | 14 | 13 | 18 | 29 | 52% | 38% |
| Other | 0 | 27 | 0 | 47 | 0% | 0% |
| None | 0 | 27 | 0 | 47 | 0% | 0% |

**
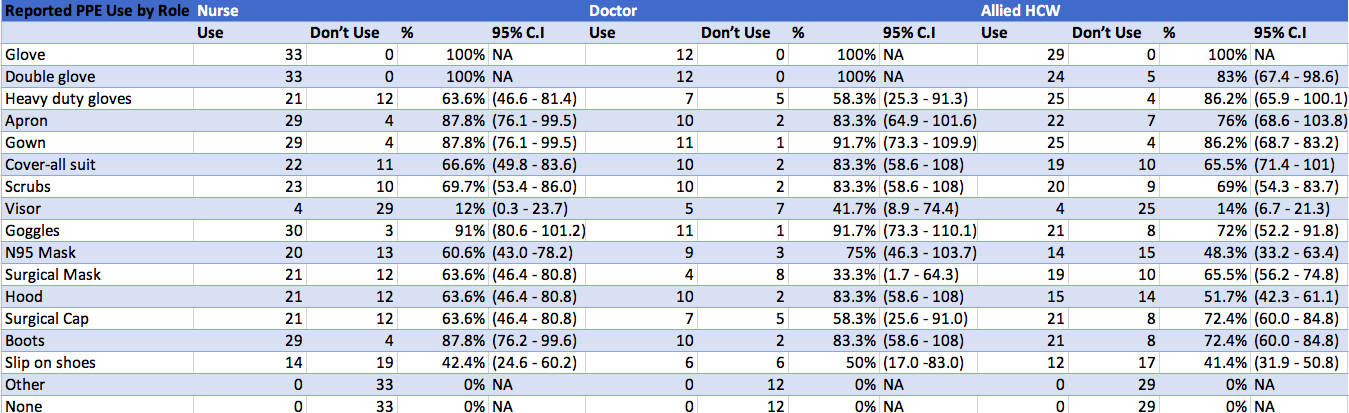
Table 2:** Reported PPE Used by health workers at all locations broken down by Occupation

**Table 3:** Reported PPE Used by HCWs at all study sites– including 95% Confidence intervals

| **Reported PPE Used by HCWs** | **Use** | **Don’t Use** | **%** | **95% C.I** |
| --- | --- | --- | --- | --- |
| Glove | 74 | 0 | 100% |  |
| Double glove | 69 | 5 | 93.2% | (87.3 - 99.1) |
| Heavy duty gloves | 53 | 21 | 71.6% | (61.1 - 82.1) |
| Apron | 61 | 13 | 82.4% | (73.5 - 91.2) |
| Gown | 65 | 9 | 87.8% | (80.2 - 95.4) |
| Cover-all suit | 51 | 23 | 68.9% | (58.1 - 79.7) |
| Scrubs | 53 | 21 | 71.6% | (61.1 - 82.1) |
| Visor | 13 | 61 | 17.6% | (8.7 - 26.5) |
| Goggles | 62 | 12 | 83.8% | (75.2 - 92.4) |
| N95 Mask | 43 | 31 | 58.1% | (46.6 - 69.6) |
| Surgical Mask | 44 | 30 | 59.6% | (48.2 - 71.1) |
| Hood | 46 | 28 | 62.2% | (50.9 - 73.5) |
| Surgical Cap | 49 | 25 | 66.2% | (55.2 - 77.2) |
| Boots | 60 | 14 | 81.1% | (72.0 - 90.2) |
| Slip on shoes | 32 | 42 | 43.2% | (31.6 - 54.8) |
