# Supplementary material for: A mixed-methods analysis of personal protective equipment used in Lassa fever treatment centres in Nigeria
Source: Infect Prev Pract. 2021 Aug 3;3(3):100168. doi: 10.1016/j.infpip.2021.100168 (PMC8367797; doi:10.1016/j.infpip.2021.100168)
Supplement: Multimedia component 3 [file mmc3.docx]

**Appendix 3 Results of Observations in practise, presented by location and combined**

**Figure 3:** Observed PPE being used across 2 Lassa Treatment Centres


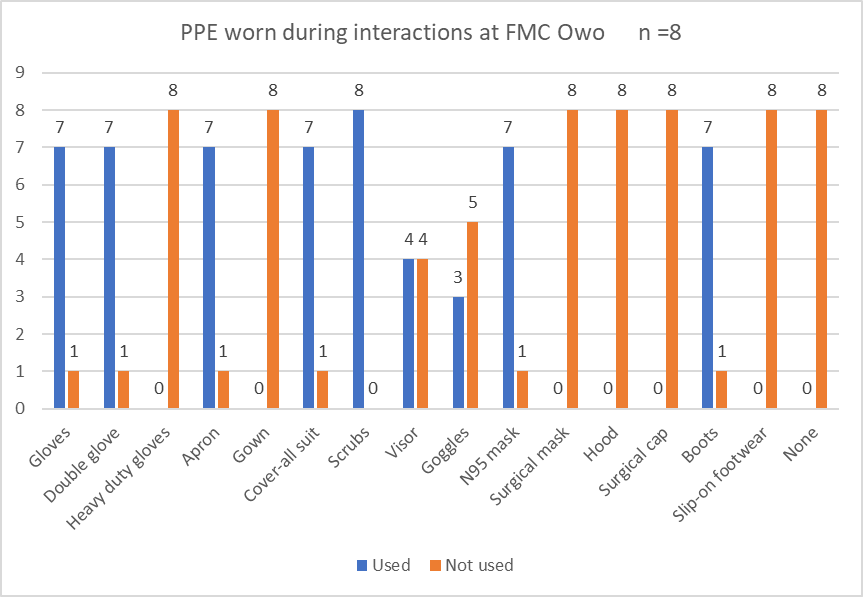


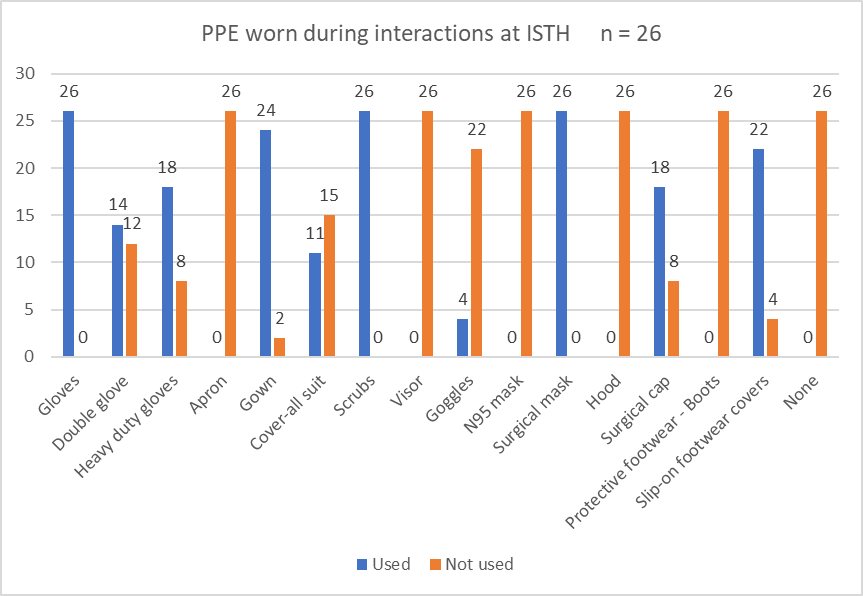


**Table 1:** PPE worn during all observations combined – including 95% Confidence intervals

| **PPE worn via Observations** | **Used** | **Not used** | **%** | **95% C.I** |
| --- | --- | --- | --- | --- |
| Gloves | 33 | 1 | 97% | (91.1 - 102.9) |
| Double glove | 21 | 13 | 61.8% | (44.7 - 78.9) |
| Heavy duty gloves | 18 | 16 | 52.9% | (35.1 - 70.7) |
| Apron | 7 | 27 | 20.6% | (6.3 - 34.9) |
| Gown | 24 | 10 | 70.6% | (54.5 - 86.8) |
| Cover-all suit | 18 | 16 | 52.9% | (35.2 - 70.6) |
| Scrubs | 31 | 3 | 91.2% | (85.3 - 97.1) |
| Visor | 4 | 30 | 11.8% | (0.3 - 23.1) |
| Goggles | 7 | 27 | 20.6% | (6.3 - 34.9) |
| N95 mask | 7 | 27 | 20.6% | (6.3 - 34.9) |
| Surgical mask | 26 | 8 | 76.5% | (61.5 - 91.5) |
| Hood | 0 | 34 | 0% | NA |
| Surgical cap | 18 | 16 | 52.9% | (35.2 - 70.6) |
| Protective footwear - Boots | 7 | 27 | 20.6% | (6.3 - 34.9) |
| Slip-on footwear | 22 | 12 | 64.7% | (47.8 - 81.6) |
