# Supplementary material for: A mixed-methods analysis of personal protective equipment used in Lassa fever treatment centres in Nigeria
Source: Infect Prev Pract. 2021 Aug 3;3(3):100168. doi: 10.1016/j.infpip.2021.100168 (PMC8367797; doi:10.1016/j.infpip.2021.100168)

**Appendix 4**

Topic Guide for interviews,

Themes and subthemes from Qualitative Data

**Fig 1. Topic Guide: used to guide discussion during the in depth interviews**

| **Core Questions** | **Follow on Questions/ prompts** |
| --- | --- |
| Can you tell me briefly a little about your role and experience with Lassa? |  |
| How has yours and your colleagues understanding of the transmission of Lassa changed over the years? | What do you feel are the reasons for this? Lassa experience, Fear, stigma,  Ebola experience |
| What has informed this understanding of the transmission of Lassa? | Eg formal training, research, on the job learning |
| When would you suspect Lassa as opposed to other diseases and how does this change behaviours? | Eg high risk occupations, during the season, |
| Do you treat patients differently if they are displaying particular symptoms ( like vomiting and diarrhoea) or do you take different precautions? | Change in the amount or type of contact, less or extra PPE? |
| Are there other situations (not related to symptoms) that you feel present a higher risk of transmission. | Do you think some patients present a higher risk of transmission? Eg to care givers, dead body management |
| Do you think that full (Ebola style) PPE is indicated at all times when working with patients with Lassa? | Do you do a formal / informal risk assessment? |
| Have you had any near misses or problems with PPE? | Have you ever had an exposure incident? |
| Do you use the NCDC guidelines for IPC for VHFs in your work? | Are you aware of these guidelines? |
| Do you ever find it easy or difficult adhere to the NCDC IPC guidelines for VHFs | What other things inform your understanding of how and when to use PPE  If easy or difficult, what makes it so. Eg logistics and supply, training, climate?  What adaptations (if any ) have you made, eg procedure or equipment?  How do you try to overcome difficulties? |
| Do you have any suggestions or advice for improving the guidelines? | Shorter/ specific for Lassa/clearer language elation to PPE |
| Please list all the things that you feel you need to do in your opinion to protect yourself or your colleagues from Lassa | What do you feel has protected you so far? |
| Which of these is most important?  / rank in order or most to least important and please give your reason why. | Rank these in order of importance |
| Do you have any final comments? Or experiences you would like to share? |  |
| Do you have any suggestion for other people who may want to talk to us and may have an perspective to share on this issue. |  |

**Fig 2. Themes and Sub themes:** Derived from the interview transcripts and grouped by the study team during analysis


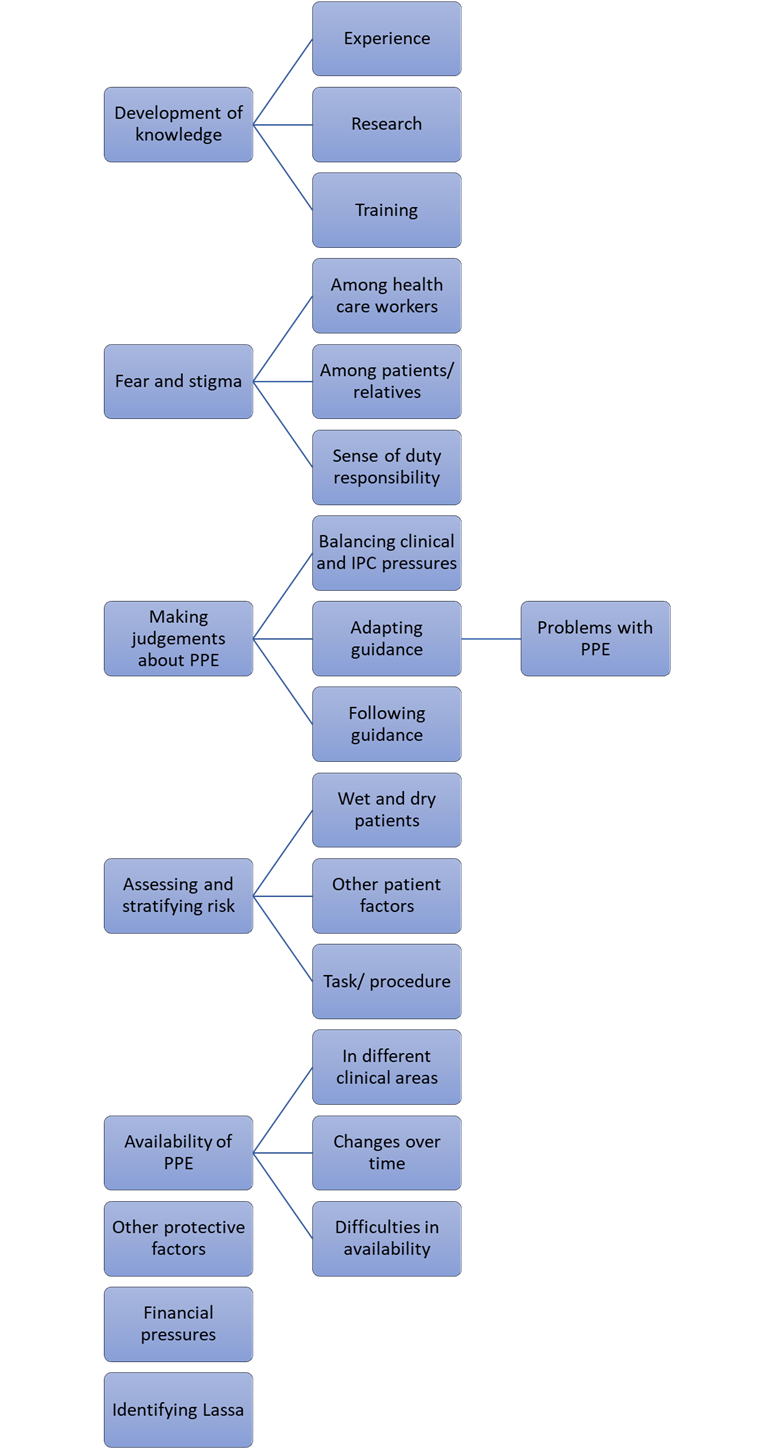

Supplement: Multimedia component 4 [file mmc4.docx]
